# microRNA-149 targets caspase-2 in glioma progression

**DOI:** 10.18632/oncotarget.8506

**Published:** 2016-03-30

**Authors:** Xiaokun Shen, Jie Li, Wenfeng Liao, Jiwen Wang, Huanjun Chen, Yanli Yao, Houbao Liu, Kan Ding

**Affiliations:** ^1^ Glycobiology and Glycochemistry Laboratory, Shanghai Institute of Materia Medica, Chinese Academy of Sciences, Shanghai 201203, China; ^2^ Department of General Surgery, Zhongshan Hospital, General Surgery Institute, Fudan University, Shanghai 200032, China

**Keywords:** glioma, microRNA-149, caspase-2, p53

## Abstract

Malignant gliomas are the most common form of intrinsic primary brain tumors worldwide. Alterations in microRNAs play a role in highly invasive malignant glioma, but detail mechanism still unknown. In this study, the role and mechanism of microRNA-149 (miR-149) in glioma are investigated. We show that miR-149 is expressed at substantially higher levels in glioma than in normal tissues. Stable overexpression of miR-149 augments potent prosurvival activity, as evidenced by promotion of cell viability, inhibition of apoptosis, and induced xenografted tumor growth *in vivo*. We further show that Caspase-2 is identified as a functional target of miR-149 and expression of caspase-2 is inversely associated with miR-149 *in vitro*. In addition, miR-149 promotes tumor survival in the U87-MG and A172 cell lines and it targets caspase-2 via inactivation of the p53 and p21 pathways. There results support a special role for miR-149 by targeting Caspase-2 to impact on p53 signaling pathway. We speculate that miR-149 has distinct biological functions in p53 wild type cells and p53 mutation cells, and the mechanisms involved remain to be explored in future. Our study suggests that targeting miR-149 may be a novel therapy strategy for treating p53 wild type glioma tumors in humans.

## INTRODUCTION

Malignant gliomas are the most common form of intrinsic primary brain tumors worldwide. They are highly invasive and are considered to be among the deadliest of human cancers [1, 2]. Based on histopathological features, gliomas are classified on a scale of I to IV, according to their degree of malignancy [3, 4]. The biologic behavior of different tumor grades can be markedly different and thus determines clinical outcome. Grade I and grade II tumors are low-grade malignancies that may undergo quite long clinical courses. However, grade III and grade IV tumors are high-grade and often lead to death within a few years. Clinically, glioma treatment includes surgery, radiotherapy and chemotherapy [5, 6]. Although significant advances in our understanding of molecular events and the development of novel therapeutic modalities, the overall prognosis remains poor for glioma patients.

Multiple mechanisms may account for the refractory nature of gliomas. Most high-risk glioblastoma multiforme (glioma grade IV) respond initially to cytotoxic therapy and local radiotherapy, however, relapses frequently occur. In addition, glioblastoma multiforme display high levels of drug resistance, which often correlate with the intensity of therapy [7–9]. Better understanding the mechanism of glioma pathogenesis and drug resistance is of great clinical significance. A large class of small, nonprotein-coding RNAs termed microRNAs (miRNAs) has been found to be a new class of gene regulators. These molecules have been shown to control fundamental cell functions, including proliferation, apoptosis and differentiation. Consequently, it has been suggested that alterations in miRNAs may play a role in carcinogenesis [10, 11]. miRNAs exert regulatory functions by binding to complementary regions in the 3′-untranslated region (UTR) of their target mRNA transcripts, which results in the posttranscriptional modification or degradation of its target RNA [12, 13]. miR-149 is one of miRNAs that have been reported to be involved in cancer. It was found to be a tumor suppressor in several cancers, including lung, colorectal and breast cancer [14–17]. Although several studies have been conducted on miR-149 and gliomas, it's not clear what exactly the function of miR-149 is and which is the target signaling regulated by miR-149 [18–20].

The current study was performed to better understand the role of miRNA-149 and its mechanism in the progression of glioma. Upregulation of miR-149 expression was found in malignant glioma tissues as compared to benign tissues. A series of *in vitro* and *in vivo* experiments was then conducted to illustrate the simulative role of miR-149 in glioma. Besides, the involvement of miR-149 and Caspase-2 in the resistance to temozolomide or cisplatin in glioma was revealed.

## RESULTS

### miR-149 was different expressed in human glioma tissues

**Figure 1:**
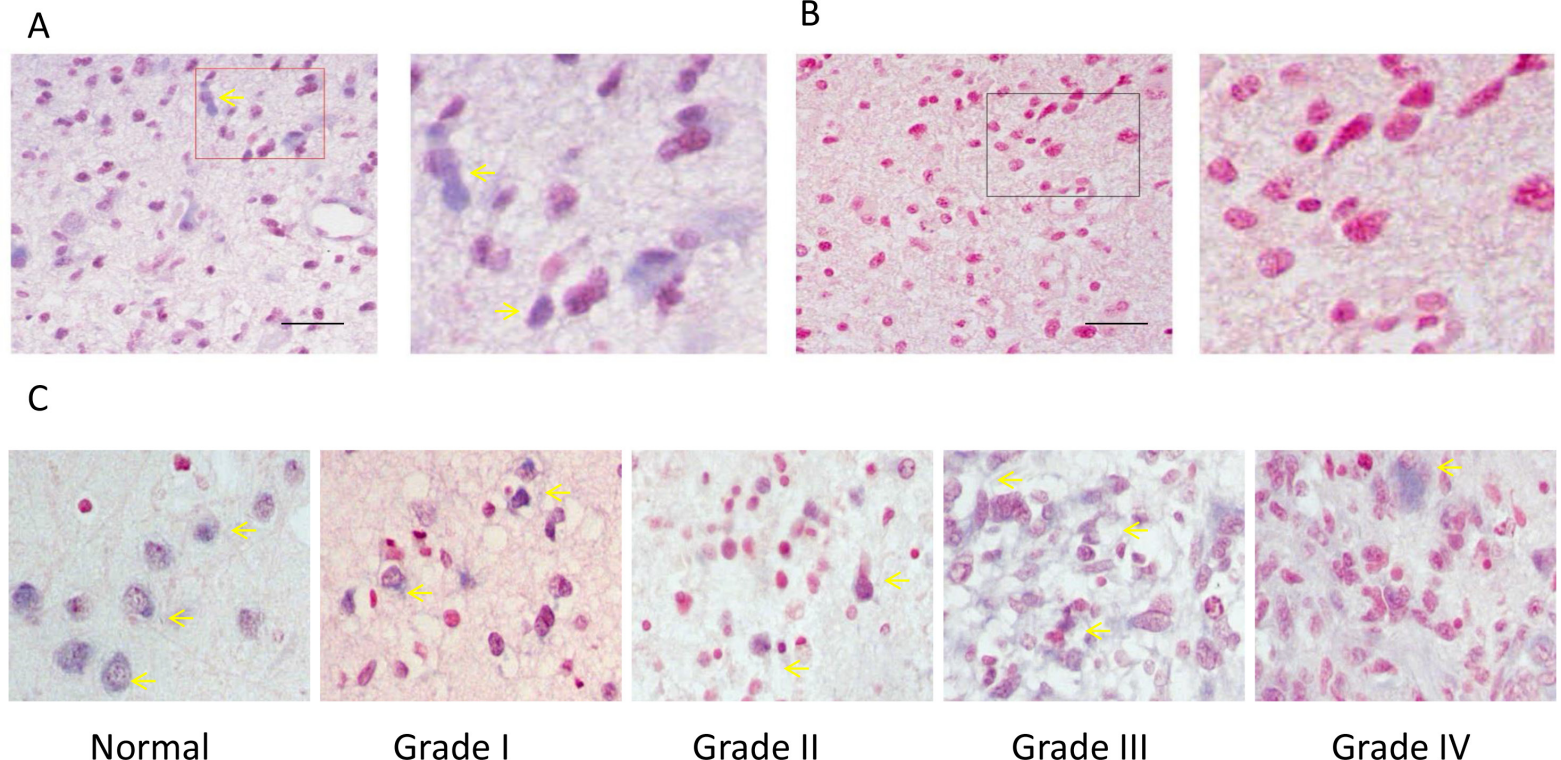
ISH analysis of miR-149 expression in normal brain tissue and in tissues of different grades of glioma carcinoma (**A**) A representative example of a glioma tissue section showing cytoplasmic staining in the vast majority of tumor cells. The right image is the enlarged image of the red box part on the left. (**B**) The no-oligonucleotide detection probe staining (control for A). (**C**) Representative images of ISH staining of miR-149 in normal brain tissue, low-grade glioma carcinoma (grades I and II), and high-grade glioma carcinoma (grades III and IV). Expression of miR-149 is moderate (+) in all pictures. Pictures were taken at 200× magnification.

In order to study the role of miR-149 in human glioma, *in situ* hybridization was performed to examine the expression level of miR-149 in normal and glioma tissues. Altogether 82 clinical samples (72 glioma samples and 10 normal brain tissues) were examined and *in situ* hybridization results showed miR-149 mainly localized in the nucleus and cytoplasm (Figure 1). The glioma sections examined included four different tumor grades: Grade I (*n* = 7), Grade II (*n* = 47), Grade III (*n* = 9) and Grade IV (*n* = 9). Analysis of the hybridization densities revealed that ratio of miR-149 positive expression in glioma tissue (79.2%) was higher than that in normal tissue (60.0%), and the difference is statistically significant, demonstrating that (P < 0.001) (Table 1). Besides, there was a difference in the ratio of miR-149 positive-expression among different grades of glioma malignancy (*p* < 0.05). In lower-grade (grades 1 and 2) glioma sections, 20.4% (11 of 54), and 51.9% (28 of 54) of the samples had moderate or low staining, respectively. While in grades 3 and 4 glioma sections, 5.6% (1 of 18) and 94.4% (17 of 18) of the samples had moderate or low staining, respectively. These results indicate that the expression level of miR-149 is positive associated with the glioma malignancy grade.

**Table 1:** Relationship between miR-149 expression and clinical pathological factors in glioma cancer

Variable	Patients	negative	positive			*p* value
			+	++	+++	
Gender						
Male	50	13 (26%)	28	8	1	0.3152 ^a^
Female	32	6 (18.8%)	19	7	0	
Age						
< = 40	36	5 (13.9%)	23	7	1	0.1135 ^a^
> 40	46	14 (30.4%)	24	8	0	
Pathological stage						
normal	10	4 (40%)	2	3	1	0.0084 ^a^
tumor	72	15 (20.8%)	45	12	0	
1	7	0 (0.0%)	4	3	0	0.0114 ^a^
II	47	15 (31.9%)	24	8	0	
III	9	0 (0.0%)	8	1	0	
IV	9	0 (0.0%)	9	0	0	

a Two-sided Fisher's Exact Test.

### miR-149 stimulated U87 MG cell growth

**Figure 2:**
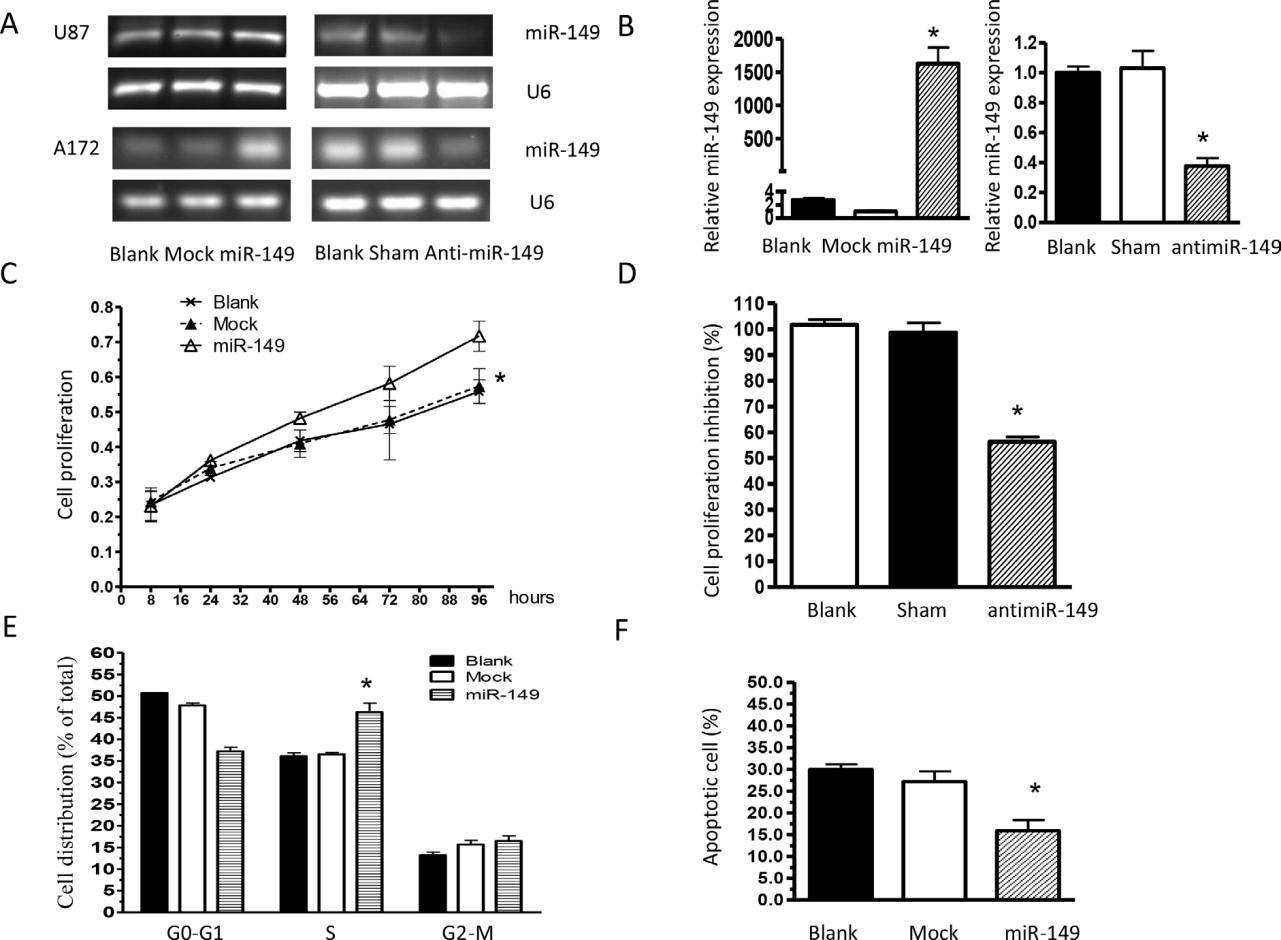
miR-149 stimulates glioma cell growth *in vitro* (**A**) miR-149 relative expression measured by RT-PCR in U87-MG and A172 cell lines. (**B**) Quantitative real-time PCR analysis of miR-149 expression. Data were normalized to U6 control. (**C**) Proliferation of miR-149-expressing U87-MG cells was significantly increased compared to the vector group. (**D**) U87-MG cell proliferation was inhibited after miR-149 knockdown. (**E**) Cell cycle analysis showing an increase in the S phase of U87-MG cells expressing miR-149. Graphs represent the mean ± SD for each experimental group. (**F**) MiR-149 dictates resistance of U87-MG to temozolomide-induced apoptosis. *p < 0.05; **p < 0.01.

To study the role of miR-149 in glioma cell proliferation, U87 MG cells stably expressing miR-149 were generated and over expression of miR-149 were confirmed by miRNA qRT-PCR analysis (Figure 2A, 2B). U87-MG cells expressing miR-149 exhibited statistically significant promotion of cell proliferation (Figure 2C) compared with vector control and blank control cells. Cell cycle analysis revealed a statistically significant increase in the S-phase of miR-149 overexpressed cells compared with those in control group (Figure 2E). MiR- 149 effects were then confirmed by knocking down miR-149 expression via infecting U87-MG cells with antisense oligonucleotide. RT-PCR and qRT-PCR results showed the miR-149 level was markedly reduced by anti-miR-149, (Figure 2A, 2B) and as shown in Figure 2D, anti-miR-149 subsequently inhibited U87-MG cell proliferation.

### Caspase-2 was verified as a functional target of miR-149

**Figure 3:**
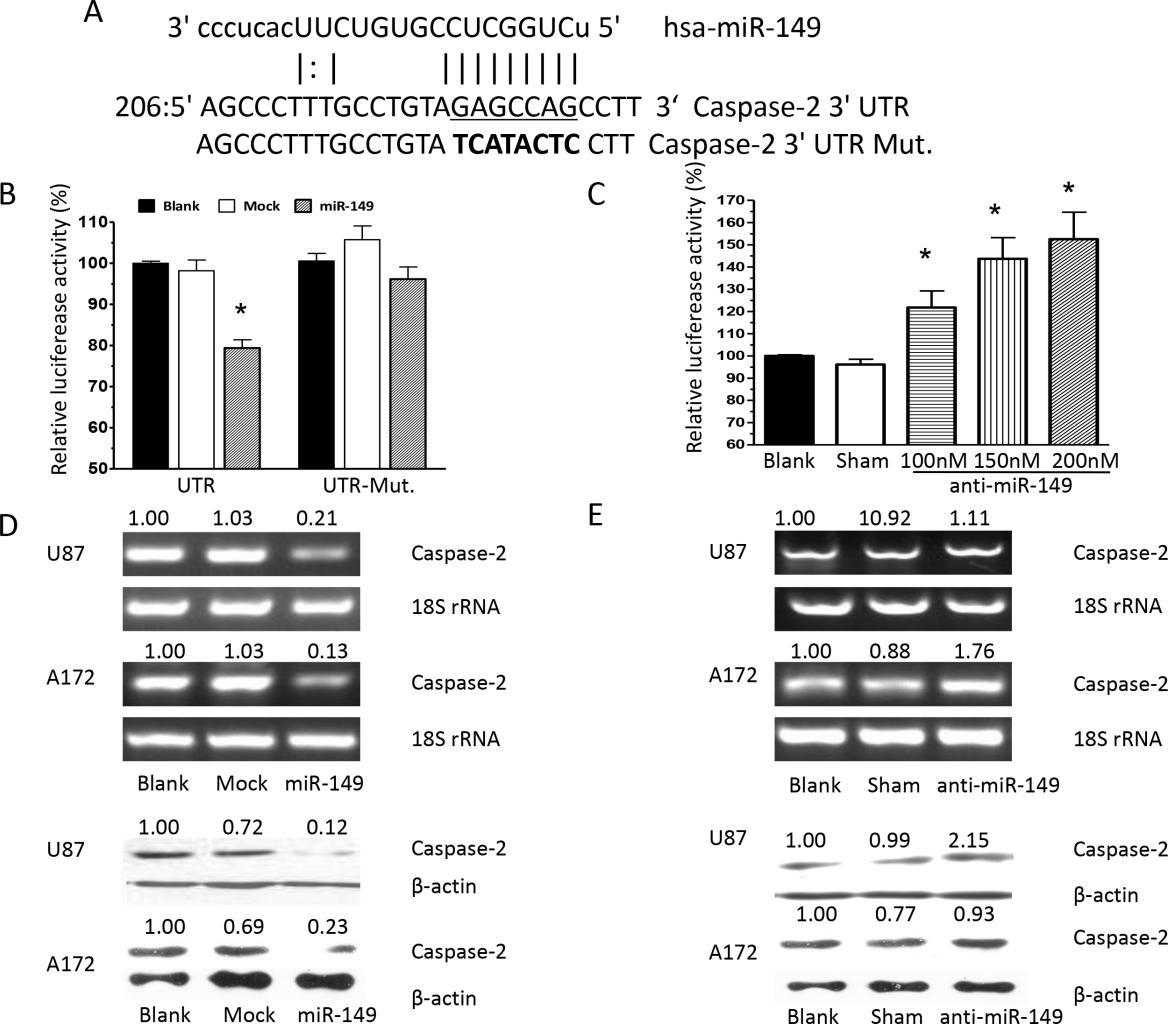
Caspase-2 is a functional target of miR-149 in U87-MG and A172 cells (**A**) Alignment of potential miR-149-binding sites in the 3′UTR of the caspase-2 mRNA. Schematic shows the 3′UTR sequences of mature miR-149 and the seed mutant region. The seed 3′UTR sequence of mature miR-149 binding is underlined. (**B**) Luciferase activity assays showing decreased reporter activity after co-transfection of either wild type caspase-2–3′UTR or it's mutated 3′UTR with miR-149 in U87-MG cells. (**C**) HEK293 cells co-transfected with caspase-2–3′UTR and anti-miR-149 showing increasing reporter activity that is concentration-dependent. (**D**) Western blot and RT-PCR analysis showing miR-149 suppression of caspase-2 expression in U87-MG and A172 cell lines. (**E**) Caspase-2 expression was enhanced after miR-149 inhibition in U87-MG and A172 cell lines. *p < 0.05; **p < 0.01.

To identify potential effectors of miR-149, algorithms were used and caspase-2 was identified as a putative target. Sequence alignment demonstrated that the seed sequence of miR-149 is complementary to the 3′UTR of caspase-2 (Figure 3A). Next, we constructed a luciferase reporter vector containing the 3′UTR of caspase-2, and transient cotransfected it along with miR-149 into HEK293 cells. MiR-149 was observed to fuse to the caspase-2 3′UTR and significantly reduced the activity of luciferase reporter gene. Besides, miR-149 cotransfection showed no effect on the activity of the reporter containing mutant caspase-2 3′UTR (Figure 3B), which suggested that miR-149 specifically targeted caspase-2 3′UTR. Furthermore, to confirm the binding and target effect, caspase-2 3′UTR was then cotransfected with anti-miR-149 and it showed does-dependently increase in the luciferase activity, implying that caspase-2 was indeed a target of miR-149 (Figure 3C). To determine whether miR-149 could functionally inhibit caspase-2 expression, caspase-2 expression was examined in miR- 149 expressed U87-MG cells. Caspase-2 mRNA and protein expression were significantly reduced in cells expressing miR- 149 (Figure 3D). Besides, caspase-2 was substantially increased at the mRNA and protein level after anti-miR-149 transfection in U87-MG cells (Figure 3E). To confirm the targeting of miR-149 on caspase-2 expression, we employed A172 cell line, another cultured human glioblastoma cell line. We observed miR-149 inhibited caspase-2 expression significantly while anti-miR-149 increased the caspase-2 expression in A172 cells (Figure 3D, 3E). The results proofed that caspase-2 was a target of miR-149.

**Figure 4:**
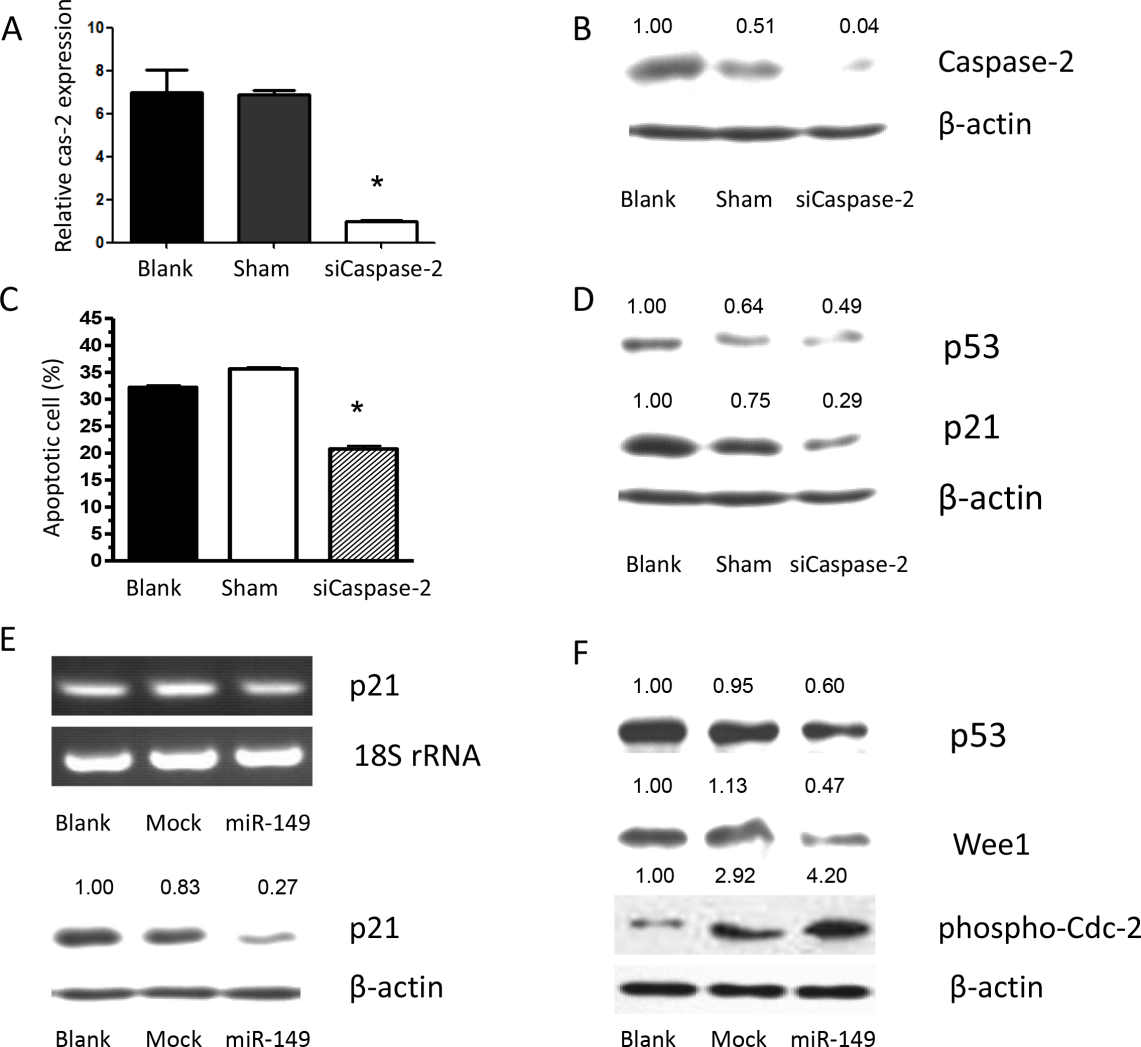
Caspase-2 deficiency caused by miR-149 expression contributes to temozolomide-induced apoptosis resistance via p21 and p53 inhibition (**A**) RT-PCR shows relative caspase-2 expression after caspase-2 knockdown by its siRNA. (**B**) Western blot shows relative caspase-2 expression after caspase-2 knockdown by its siRNA. (**C**). Apoptosis induced by temozolomide was augmented by caspase-2 expression. Proliferation assay showing inhibition of proliferation after caspase-2 over expression in U87-MG cells. (**D**) siRNA of caspase-2 showed down regulating p53 and p21 expression. (**E**) Relative p21 expression were decreased after miR-149 expression in U87-MG cells. (**F**) miR-149 expression down-regulated p53, Wee1 expression, while phosphorylation of CDC-2 was activated in U87-MG cells.*p < 0.05; **p < 0.01.

To further explore the role of caspase-2 as a functional target of miR-149, U87 MG cells were transfected with caspase-2, the effects on gene expression and U87 MG cell survival were examined. Overexpression of caspase-2 was confirmed by RT-PCR and Western blot analysis (Supplementary Figure S1A). Caspase-2 overexpression induced apoptosis and significantly suppressed proliferation of U87 MG cells compared with control cells (Supplementary Figure S1B). To confirm the effect of caspase-2 on cell proliferation, U87 MG cells were infected with siRNAs to knock down caspase-2 expression. Scrambled siRNA was used as a control. The caspase-2 mRNA and protein levels were markedly reduced by siCaspase-2 (Figure 4A, 4B). Knockdown of caspase-2 significantly suppressed cell apoptosis induced by temozolomide (Figure 4C). Similarly, miR-149 overexpression increased temozolomide-induced cell apoptosis (Figure 2F). Inhibition of miR- 149 suppressed the growth of glioma cells. These results indicate that the effects of miR-149 on U87 MG cell proliferation are mediated largely by the inhibition of caspase-2 expression.

### miR-149 downregulated caspase-2 and p53 expression *in vitro*

To elucidate the mechanism by which miR-149 increases glioma cell growth, the expression of p53 and p21 was examined in miR-149 overexpressing cells. The mRNA and protein levels of p53 and p21 were significantly downregulated by miR-149 (Figure 4E). The potential effects of miR-149 on the cell cycle kinases, which are largely controlled by p53 and p21, were then examined due to their importance in regulating the transition between each of the cell cycle phases. Central in regulating the transition between the G2 and M phases is the Wee1-like protein kinase (Wee1). Wee1 negatively stimulates entry into mitosis by phosphorylating the Tyr15 residue of Cyclin Dependent Kinase 1 (CDK1, also known as CDC2), thus inactivating the CDK1/cyclin B complex and arresting the cell cycle. miR-149 overexpression in U87 MG cells resulted in a statistically significant downregulation of the biomarker, Wee1 (Figure 4F). Subsequently, the phosphorylation level of the tyr15 residue of CDK1 was induced in miR-149 overexpressing cells (Figure 4F). Together with Figure 3, these findings suggest that miR-149 suppresses the p53 and p21 pathway and promotes cell proliferation. p53 stimulates a wide network of signals that act through two major apoptotic pathways. Intriguingly, key subsets of the Bcl2 family genes are p53 targets. Phosphorylation of MDM and BAD were induced in miR-149 overexpressing cells unpublished data. To confirm the effects of miR-149 on downstream gene expression and that cell proliferation is mediated by its inhibition of caspase-2 expression, the effect of caspase-2 knockdown on p53 and p21 expression was investigated. The levels of p53 and p21 were downregulated by the knockdown of caspase-2 (Figure 4D). In addition, small interfering RNAs against p53 and p21 in U87 MG cells reduced caspase-2 expression and led to inhibition of U87 MG cell apoptosis, respectively unpublished data. These results indicate that the effects of miR-149 on downstream p53 and p21 expression, the concomitant decrease in Wee1 expression and CDK1 phosphorylation and the promotion of cell proliferation were mediated largely by the inhibition of caspase-2 expression by miR-149.

### miR-149 promoted xenografted glioma cells growth and proliferation by down-regulating caspase-2 expression

**Figure 5:**
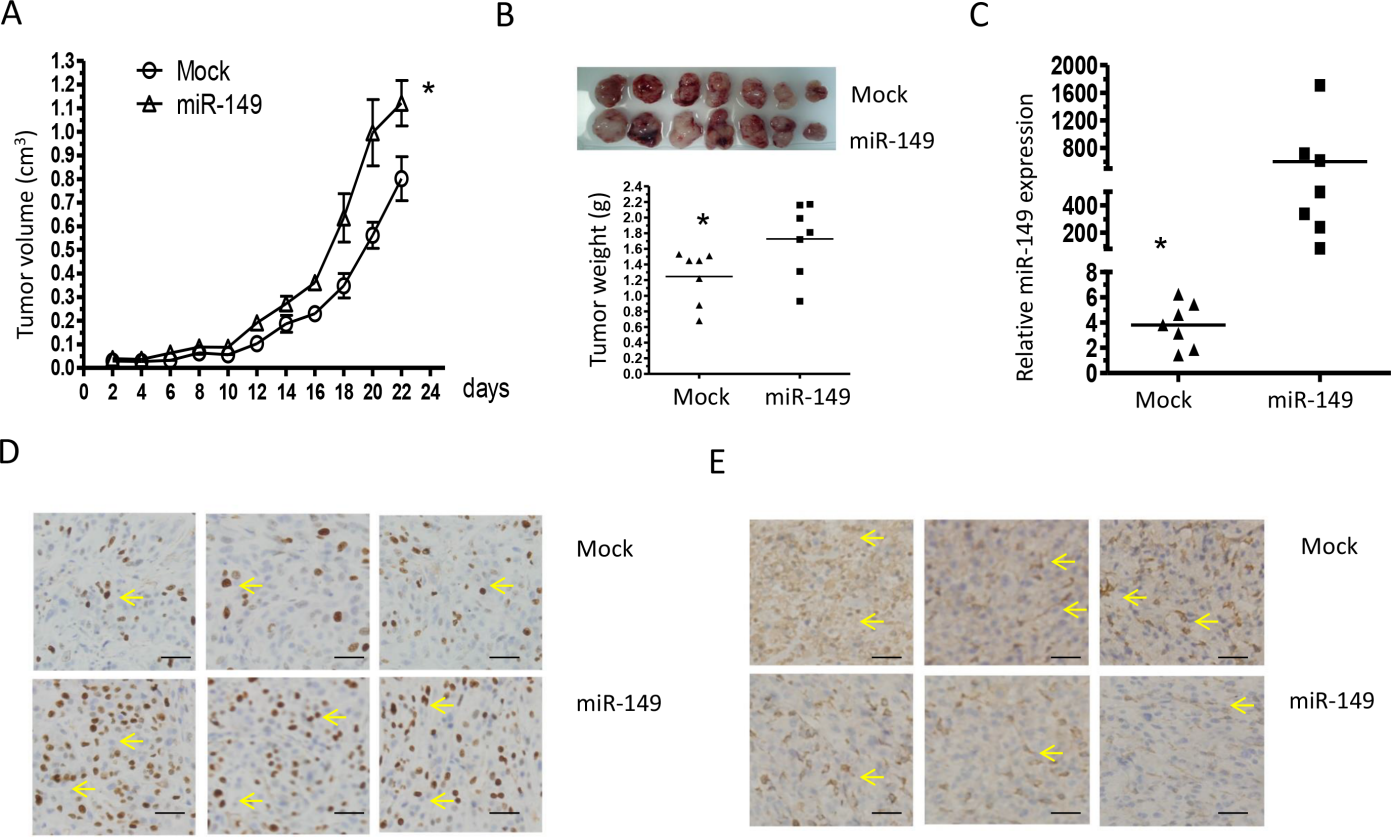
Elevated miR-149 expression in U87-MG cells promotes tumor growth and proliferation *in vivo* (**A**) Growth curves of miR-149 stably expressed U87-MG tumors. The volume of the tumors was measured every two days and each data point represents the mean ± SD in 7 mice. (**B**) Primary tumors removed from the mice and tumor weights showing the promotion of miR-149 in cell growth. (**C**) Quantitative miR-149 expressions in xenografts. (**D**) Immunohistochemistry in sections derived from U87-MG tumors. Sections were incubated with Ki67 antibody and caspase-2 antibody for detection of cell proliferation and caspase-2 expression, respectively. Yellow arrow indicates typical staining. (**E**) Sections derived from tumors with anti-antibodies for p53 and p 21. Representative fields are shown. *p < 0.05; **p < 0.01.

To provide evidence that miR-149 is indeed important for glioma development *in vivo*, a subcutaneously xenografted glioma model was established in nude mice. Tumor volumes were estimated in the subcutaneous model every 2 days post-implantation (Figure 5A). MiR-149 infected cells grew significantly faster than empty vector infected cells. Twenty-two days after implantation, the tumor-bearing mice did not show any deterioration in physiological condition. The tumors were collected after euthanizing the animals at the end of *in vivo* tumor growth evaluation. Tumors formed by miR-149 infected cells were visibly larger than those formed by control cells and the mean weight of tumors formed by miR-149 overexpressing cells was heavier than that by control cells (Figure 5B). Total RNA was then extracted from miR-149 overexpressing and the empty vector xenografts. The result shows that miR-149 levels were higher in miR-149 overexpressing xenografts compared to empty vector xenografts by realtime PCR (Figure 5C). Immunohistochemistry showed that the number of proliferation marker Ki67 in miR-149 tumor sections was significantly higher compare to control tumor sections, indicating that proliferation was enhanced by miR-149 *in vivo* (Figure 5D). Caspase-2 expression was also suppressed in the miR-149 xenografts, which is consistent with the previous *in vitro* results (Figure 5D). In addition, p21 and p53 expression levels were suppressed in miR-149 overexpressing tumors (Figure 5E). These results confirm the role of miR-149 in glioma and the effects of miR-149 on the caspase-2-p53 pathway.

### Expression of miR-149 was inversely associated with caspase-2 in glioma tissues

**Figure 6:**
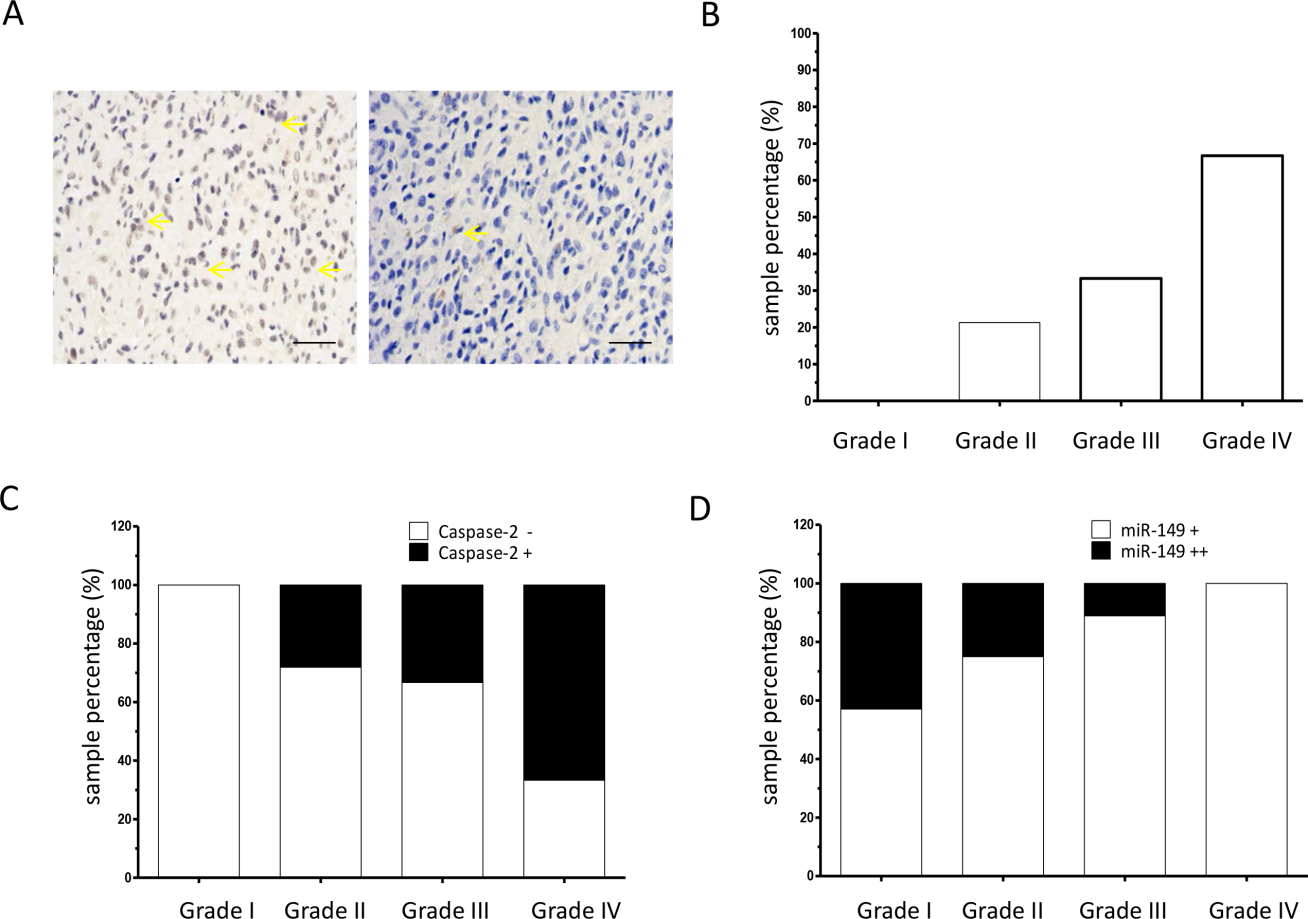
Caspase-2 expression is correlated with tumor grade in glioma (**A**) Representative images of immunohistochemical staining of caspase-2 proteins in glioma tissue sections (200 ×). Yellow arrow indicates typical staining. (**B**) Percentage of caspase-2 positive expression in low-grade (grades I and II) and high-grade (grades III and IV) glioma carcinoma specimens as analyzed by immunohistochemistry on a human glioma tissue microarray (*N* = 72). (**C**) Percentage of caspase-2 positive (+) expression in miR-149 positive glioma carcinoma subgroups (*N* = 57). (D) Immunohistochemistry showing percentage of moderate miR-149 expression (+) and strong miR-149 expression (++) in low-grade (grades I and II) and high-grade (grades III and IV) glioma carcinoma subgroups (*N* = 57). **p < 0.01.

To confirm the association between the expression of miR-149 and caspase-2, caspase-2 expression was determined at the protein level in the same panel of glioma tissues collected from clinic. Immunohistological staining of the tissues using a polyclonal caspase-2 antibody showed strong staining in the representative glioma samples (Figure 6A). The relative expression levels of different grades of glioma are shown in Figure 6B. Grade 1 glioma tissues were negative for caspase-2. However, in low-grade (grade 2) glioma sections, 21.3% samples were positive for caspase-2. In high-grade (grade 3 and 4) glioma sections, 33.3% and 66.7% of samples were positive for caspase-2, respectively (Figure 6B and Table 2). These results indicate that the expression level of caspase-2 is associated with the glioma malignancy grade. The caspase-2 level was then analyzed in the miR-149-positive subgroup (*N* = 57). The results showed that there was an inverse association between expression of caspase-2 and miR-149 (Figure 6C, Table 3). In addition, results indicate that moderate staining of miR-149 (++) was inversely associated with malignancy grade Figure 6D.

**Table 2:** Relationship between Caspase-2 expression and clinical pathological factors in glioma cancer

Variable	Patients	negative	positive	*p* value
Gender				
Male	50	36 (72.0%)	14 (28.0%)	0.8042 ^a^
Female	32	24 (75.0%)	8 (25.0%)	
Age				
< = 40	36	24 (66.7%)	12 (33.3%)	0.3160 ^a^
> 40	46	36 (78.3%)	10 (21.7%)	
Pathological stage				
normal	10	7 (70%)	3 (30.3%)	1.0000 ^a^
tumor	72	53 (73.6%)	19 (26.4%)	
1	7	7 (100%)	0 (0.0%)	0.0024 ^b^
11	47	37 (78.7%)	10 (21.3%)	
III	9	6 (66.7%)	3 (33.3%)	
IV	9	3 (33.3%)	6 (66.7%)	

a Two-sided Fisher's Exact Test.

b Mann-Whitney *U* Test.

**Table 3:** Relationship between Caspase-2 expression and tumor stage in miR-149 positive glioma cancer (N = 57)

tumor	Patients	Caspase-2		*Z* value	*p* value	*r* value	*p* value
		negative	positive				
1	7	7 (100%)	0 (0.0%)				
II	32	23 (71.9%)	9 (28.1%)				
				−2.6551	0.0079 ^a^	0.3548	0.0068 ^b^
III	9	6 (66.7%)	3 (33.3%)				
IV	9	3 (33.3%)	6 (66.7%)				

a Mann-Whitney *U* Test.

b Spearmans' correlation Test.

## DISCUSSION

Caspase-2 was the second caspase genes to be cloned and is the most evolutionarily conserved, however the function of caspase-2 has remained enigmatic [21]. On the basis of its reported proapoptotic properties, caspase-2 is considered to act as a tumor suppressor [22–24]. Reduced caspase-2 protein levels have also been reported in a meta-analysis of published data sets for Burkitt lymphoma (BL), mantle cell lymphoma (MCL), chronic lymphocytic leukemia (CLL) and hairy cell leukemia [23, 25]. Caspase-2 has also been reported to be significantly underexpressed in metastatic brain tumors [26]. In contrast, caspase-2 is constitutively expressed in most human malignant glioma cell lines [27]. To the best of our knowledge, the current study is the first one to report that there is no significant difference in caspase-2 levels between normal and glioma tissues (33.3% vs. 26.4%, *p* > 0.05). However, the results suggest that the expression level of caspase-2 is proportional depending on the glioma malignancy grade. The precise function of caspase-2 (whether its functions as an initiator or effector caspase) is still unknown. It has been suggested that caspase-2 functions as the most apical caspase when apoptosis is induced by DNA damage and cytotoxic stress [28, 29]. The involvement of caspase-2 activation in breast cancer cell apoptosis induced by various stimulations has been found [30, 31]. Several other studies have also demonstrated caspase-2 activation in various types of cancer cells following induction of apoptosis by taxanes [32, 33]. These findings indicate that caspase-2 is likely a key regulator of tumor apoptosis progression.

To our knowledge, results of the current study identify for the first time that the tumor effects of miR-149 are mediated, in large part, by inhibition of caspase-2 expression. These observations indicate that the intrinsic apoptosis machinery is active in tumors and that miR- 149 could be a potential target for selectively killing cancer cells. Based on this consideration, the expression of miR-149 could be examined in cancer patients who are refractive to chemotherapy. Finally, although caspase-2 was identified as a target of miR- 149 in U87 MG cells, it is possible that miR-149 targets other genes in distinct subsets of glioma tumors. Previous study showed miR-149 were downregulated significantly in grade I-III astrocytomas using microarrays, which is consist with our study that normal tissues have more moderate (++) or high (+++) staining with FISH.

Although the functional consequences of miRNA expression are commonly tissue- and cell type-specific, p53 regulation of its target genes also occurs in a cell type- and stimulus dependent manner. Mutations of p53 are present in more than 30% of gliomas, and constitute an early genetic event, suggesting that abnormalities of p53 are involved in development of gliomas [34, 35]. Restoring the function of wild-type p53 was found to induce apoptosis in human glioma cells expressing mutant p53, as assessed by acridine orange nuclear staining, electron microscopy and flow cytometry assays. In contrast, expression of exogenous p53 in human glioma cells that contained a wild-type p53 gene inhibited cell growth, but did not induce apoptosis. The different p53 effects suggest that there are different pathways for glioma tumorigenesis and point out the opportunity for designing more selective therapeutic strategies by taking advantage of the increased vulnerability of mutant p53 cells. It has been shown that U251 MG and U373 MG have a mutation in codon 273 of the p53 gene and cannot transactivate a reporter gene containing a p53-responsive transcriptional promoter, while the endogenous p53 gene in U87 MG cells is known to have a wild-type sequence in exons 2 through 11. Despite the clear evidence showing a prosurvival role of miR-149 in U87 MG and A172 cells (wild-type p53), miR-149 has been reported to be a tumor suppressor, which may be involved in the proliferation and invasion of U251 GM cells (p53 mutant) via blockade of AKT1 signaling and miR-149 inhibit SF126 glioblastoma cells proliferation and migration (p53 loss) [18, 20]. We speculate that miR-149 has distinct biological functions in p53 wild type cells and p53 mutation cells, although the mechanisms involved remain to be defined and need to be explored in future experiments.

In summary, this study presented evidences that miR-149 was involved in the progression of glioma. Caspase-2 is a functional target of this microRNA. Molecules in p53 signaling pathway mediated the effects of miR-149 as well. These findings suggest that targeting miR-149 may be a novel therapy strategy for treating p53 wild type glioma tumors in humans.

## MATERIALS AND METHODS

### Cell culture

The human glioma cell line, U87-MG, A172 and embryonic kidney HEK293 cells were cultured in Dulbecco's modified Eagle's medium (HyClone, U.S.A.), supplemented with 10% fetal bovine serum (Gibco, U.S.A.), 100 units/ml of penicillin and 100 ug/ml of streptomycin (Invitrogen, U.S.A.). All cells were maintained at 37°C, in a humidified atmosphere, with 5% CO_2_ in air, and subcultured every 3–5 days.

### miRNA Oligo, siRNA, cDNA construction and transfection

miR-149 antisense and scrambled control oligonucleotides were obtained from GenePharma Co., Ltd. (Shanghai, China), the siRNA directed against caspase-2 was synthesized by RiBoBio Co., Ltd. (Guangzhou, China) and transfections were carried out using Lipofectamine 2000 (Invitrogen, U.S.A.) according to the manufacturer's instructions. Briefly, U87-MG Cells (4 × 10^3^ cells/well) were seeded into the 96-well plate for 24 h before transfection. After transfection for 72 h, 5 mg/mL MTT was then added to each well and incubated for 4 h. The formazan crystals formed from MTT by the living cells were dissolved in 150 μL DMSO, and then detected by a spectrophotometer (Thermo Scientific, USA) at an absorption wavelength of 490 nm. The inhibitory ratio was calculated as ((control − sample)/control) × 100%.

The lentivirus-expressing miR-149 was constructed by Genechem Co., Ltd. and the lentivirus infection was performed according to the manufacturer's instructions. Briefly, U87-MG cells (1 × 10^5^) were cultured in a 24-well plate for 24 h. An amount of 50 μL per well was added to the plate. Lentiviral vector GFP fluorescence was detected with an Olympus IX51 digital camera microscope 48 h after the virus was added.

### RNA isolation, RT-PCR and quantitative real-time PCR

Total RNA was extracted from frozen primary tumors and/or cell lines using TRIzol reagent (Invitrogen, U.S.A.). RNA (2.0 μg) was used to synthesize cDNA via moloney murine leukemia virus reverse transcriptase (TaKaRa, Japan), according to the manufacturer's instructions. Detection of mature miRNAs was performed using the miRNA Primer (Guangzhou RiboBio Co., Ltd., China), according to the manufacturer's instructions. Semi-quantitative RT-PCR was used to evaluate the caspase-2 mRNA level. The primer used was: 5′-GCAGTTTCAGC CAGAATGTG-3′ (sense) and 5′-AGGGTGACTAGAGTA CTGTGG-3′ (antisense). Quantitative real-time PCR was performed using an Applied Biosystems 7500 Fast Real-time PCR system with an SYBR Green Premix Ex Taq kit (TaKaRa, Japan). Each sample was run in triplicate and Ct was determined for the target transcripts. Caspase-2 levels were quantified using the ΔΔCt method.

### Western blot analysis

Proteins isolated from cells were separated on a 10% SDS-PAGE gel and the proteins were transferred to polyinylidene fluoride (PVDF) membranes. Blotting was carried out for caspase-2 (Cell Signaling Technology, U.S.A.) and β-actin (Sigma, U.S.A.) was used as the loading control. Secondary horseradish peroxidase antibody was detected using the ECL Western Blotting Analysis System (Pierce, U.S.A.).

### Dual luciferase reporter assays

Luciferase reporter assays were performed using the psiCHECK2–3′UTR vector. Cells were transiently cotransfected with wild-type or mutated reporter psicheck-2 plasmid and miR-149 or vector control. Firefly luciferase activities were measured 36 h after transfection using the Dual Luciferase Assay (Promega), and the renilla luciferase activity was normalized to firefly luciferase. Each sample was assayed in triplicate. Each reporter plasmid was transfected at least three times and each sample was assayed in triplicate.

### Tumor xenograft growth assay

All animal experiments were approved by the Institutional Animal Care and Use Committee and Local Ethical Board. miR-149 overexpressed stable U87-MG cells (1 × 10^6^) or empty vector U87-MG cells (1 × 10^6^) were subcutaneously injected into the mammary fat pads of 4–5-week-old BALBc nude male mice, respectively. Growth curves and tumor volumes were measured every other day. The tumor volume was determined according to the equation: V = (A × B^2^) × 0.5, where V is volume, A is length, and B is width. On the 22nd day after injection, the mice were euthanized and the tumors were harvested, weighed and photographed.

### *In situ* detection of miRNA

The miRCURY miR-149 3′-digoxigenin (DIG)-labeled probe, the 3′-DIG-labeled scrambled-miR negative control probe, the 3′-DIG labeled U6 positive control probe and the LNA-modified oligonucleotide detection probe were purchased from Exiqon (Denmark). Paraffin sections were deparaffinized in xylene and rehydrated in graded alcohols and distilled water. Sections were then treated with proteinase K (10 mg/mL) for 10 minutes at 37°C, rinsed in 0.2% glycine for 1 minute and fixed in 4% paraformaldehyde for 10 minutes at 25°C. Before hybridization, sections were prehybridized in hybridization buffer at 50°C for 2 h. The DIG-labeled LNA probe was diluted to 20 nmol/L in hybridization buffer. Slides were hybridized overnight with the diluted probe in a humidified chamber at 50°C. Sections were then washed with 2× SSC followed by 0.2 × SSC at 50°C. After a blocking step (2% sheep serum, 2 mg/mL BSA in PBST) at room temperature for 1 h, the slides were incubated with anti-DIG-AP Fab fragments (1:1000, Roche Diagnostics) at 4°C, overnight. Signals were detected by incubating sections in NBT-BCIP solution (Roche Diagnostics) for up to 4 days. Sections were imaged using the Scanscope system (Aperio) and then compared with the hematoxylin and eosin (H & E) image of the corresponding section. The abundance of miR-149 was categorized as high, moderate or low based on the intensity of staining by two different pathologists.

Tumor tissues were excised, fixed in 4% neutral paraformaldehyde, embedded in paraffin, and sectioned, immunohistochemistry analysis was done as described previously. Identification of tumor cell proliferation was performed via immunostaining using a monoclonal antibody against Ki67 at a 1:100 dilution at 4°C overnight. For caspase-2 expression detection, *in vivo*, the sections were stained using anti-caspase-2 antibody at a 1:50 dilution at 4°C overnight. Semi-quantitative image analysis of each section was employed to measure the integrated optical density using Image Pro Plus software (Media Cybernetics, U.S.A.).

### Flow cytometry

FACS analysis for cell cycle and apoptosis was done 72 h post-transfection using nuclear stain PI for cell cycle analysis or a ANNEXIN V-FITC KIT (Invitrogen, U.S.A) for apoptosis analysis, according to the manufacturer's protocol. Briefly, for the apoptosis assay, U87-MG cells were harvested by trypsinization. After washing with PBS, cells were resuspended in 500 μL binding buffer, and then stained with an Annexin V-FITC and PI solution for 30 min at room temperature in the dark. The samples were analyzed immediately using the FACS Calibur flow cytometer (BD Biosciences, U.S.A). For cell cycle analysis, after washing with PBS, the cells were fixed in ice-cold 70% (v/v) ethanol overnight. Fixed cells were resuspended in 500 μl PBS containing 50 mg/ml RNase (Sigma-Aldrich, U.S.A) and incubated at 37°C for 30 min. After staining with 50 mg/ml PI (Sigma-Aldrich, U.S.A) at 4°C in the dark for 30 min, cell cycle distribution was analyzed and the results were analyzed using Cell Quest software.

## SUPPLEMENTARY MATERIALS FIGURE


