# Supplementary material for: microRNA-149 targets caspase-2 in glioma progression
Source: Oncotarget. 2016 Mar 30;7(18):26388–99. doi: 10.18632/oncotarget.8506 (PMC5041987; doi:10.18632/oncotarget.8506)
Supplement: Supplementary file 1 [file oncotarget-07-26388-s001.pdf]

microRNA-149 targets caspase-2 in glioma progression

Supplementary Materials

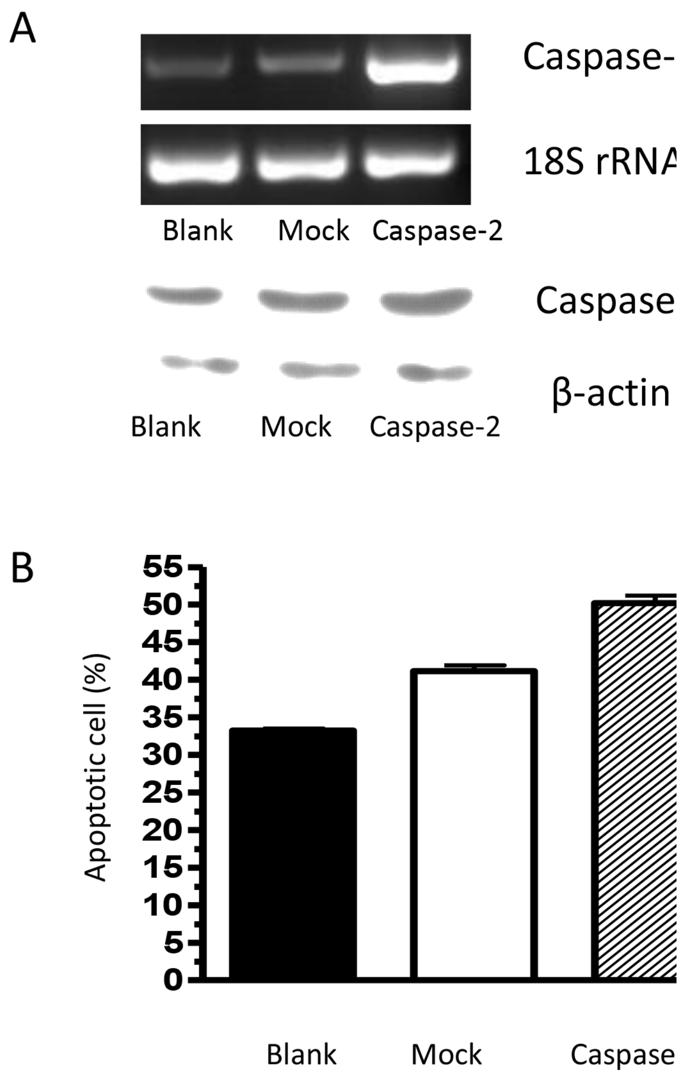

Supplementary Figure S1: Caspase-2 expression resists to cisplatin induced apoptosis. (**A**) RT-PCR and Western blot showing relative caspase-2 expression in U87-MG cells. (**B**) Apoptosis induced by cisplatin was augmented by caspase-2 expression.
